# Supplementary figures and images for: Regional fat depot masses are influenced by protein-coding gene variants
Source: PLoS One. 2019 May 30;14(5):e0217644. doi: 10.1371/journal.pone.0217644 (PMC6542527; doi:10.1371/journal.pone.0217644)

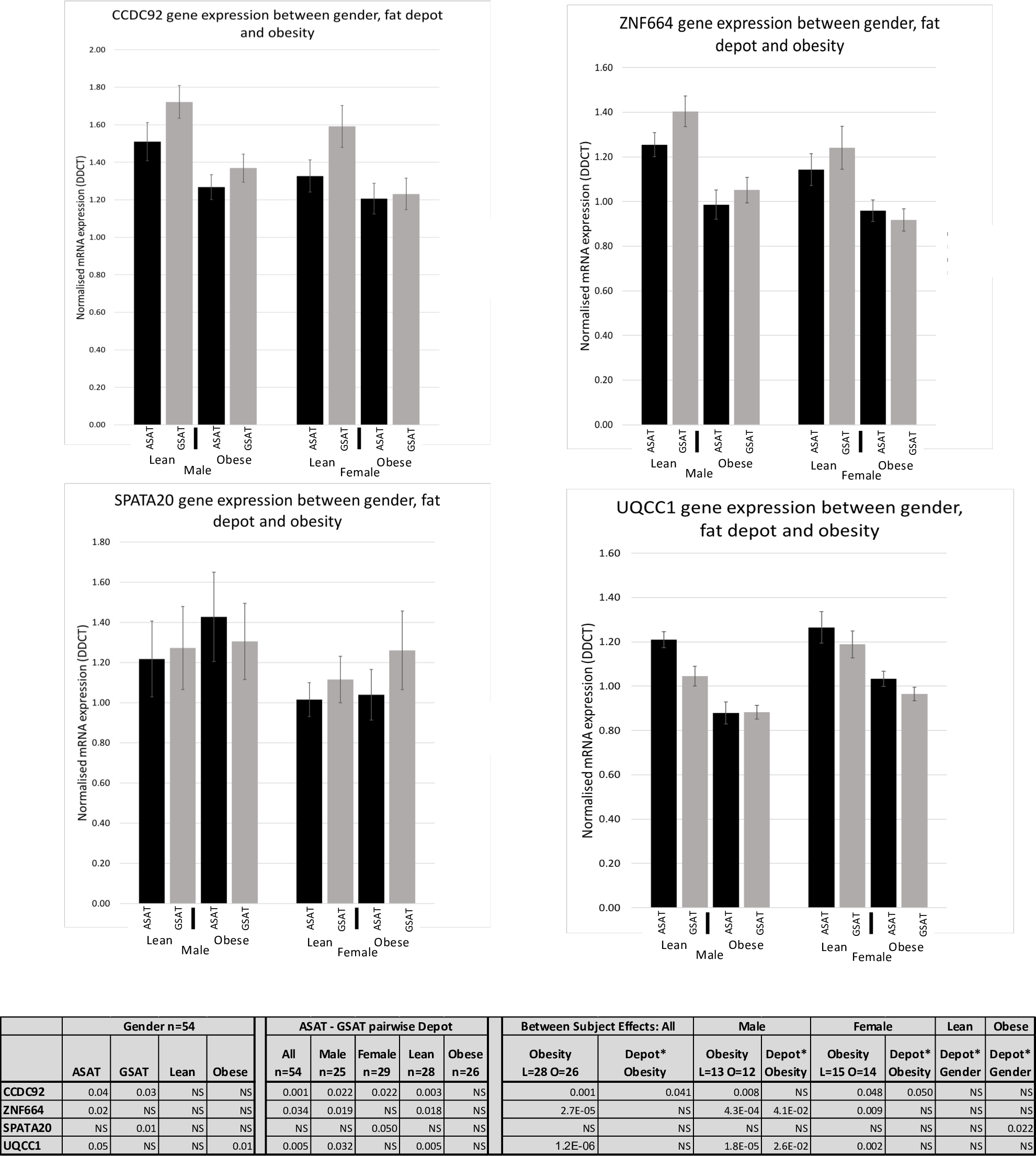

Supplement: S1 Fig — mRNA expression of the genes CCDC92, ZNF664, UQCC1 and SPATA20 across a panel of paired abdominal subcutaneous adipose tissue (ASAT) and gluteal subcutaneous adipose tissue (GSAT) cDNA samples from the Oxford Biobank. The panel consisted of 25 male and 29 female healthy individuals selected for either high or low BMI (Lean male, n = 13, age 44.5±0.9 yrs, BMI 22.7±0.3 kg/m2, fasting blood glucose 5.2±0.1 mmol/l; Obese males, n = 12, age 43.4±1.2 yrs, BMI 34.9±5.2 kg/m2, fasting blood glucose 5.6±0.1 mmol/l; Lean females, n = 15, age 44±1.0 yrs, BMI 21.2±0.2 kg/m2, fasting blood glucose 4.8±0.1 mmol/l; Obese Females, n = 14, age 44±1.0 yrs, BMI 33.6±0.6 kg/m2, fasting blood glucose 5.2±0.1 mmol/l–data expressed as mean ±SEM). Data are shown as the mean ± SEM DDCt values (normalized to the geometric mean of the endogenous control genes PPIA, PGK1, IPO8 and PSMB6) as described previously[39, 41]. A multivariate general linear model was used to test for statistical significance between gender, fat depots and obesity and to assess interactions. P-values are presented in the shaded box, NS: non-significant. There were small but significant differences in expression of CCDC92, ZNF664 and UQCC1 between fat depots in lean individuals but this difference was lost and expression was significantly reduced, in obese individuals. This is in keeping with a general quiescent state observed in transcripts associated with adipocyte metabolic activity in obesity. (TIF) [file pone.0217644.s001.tif]

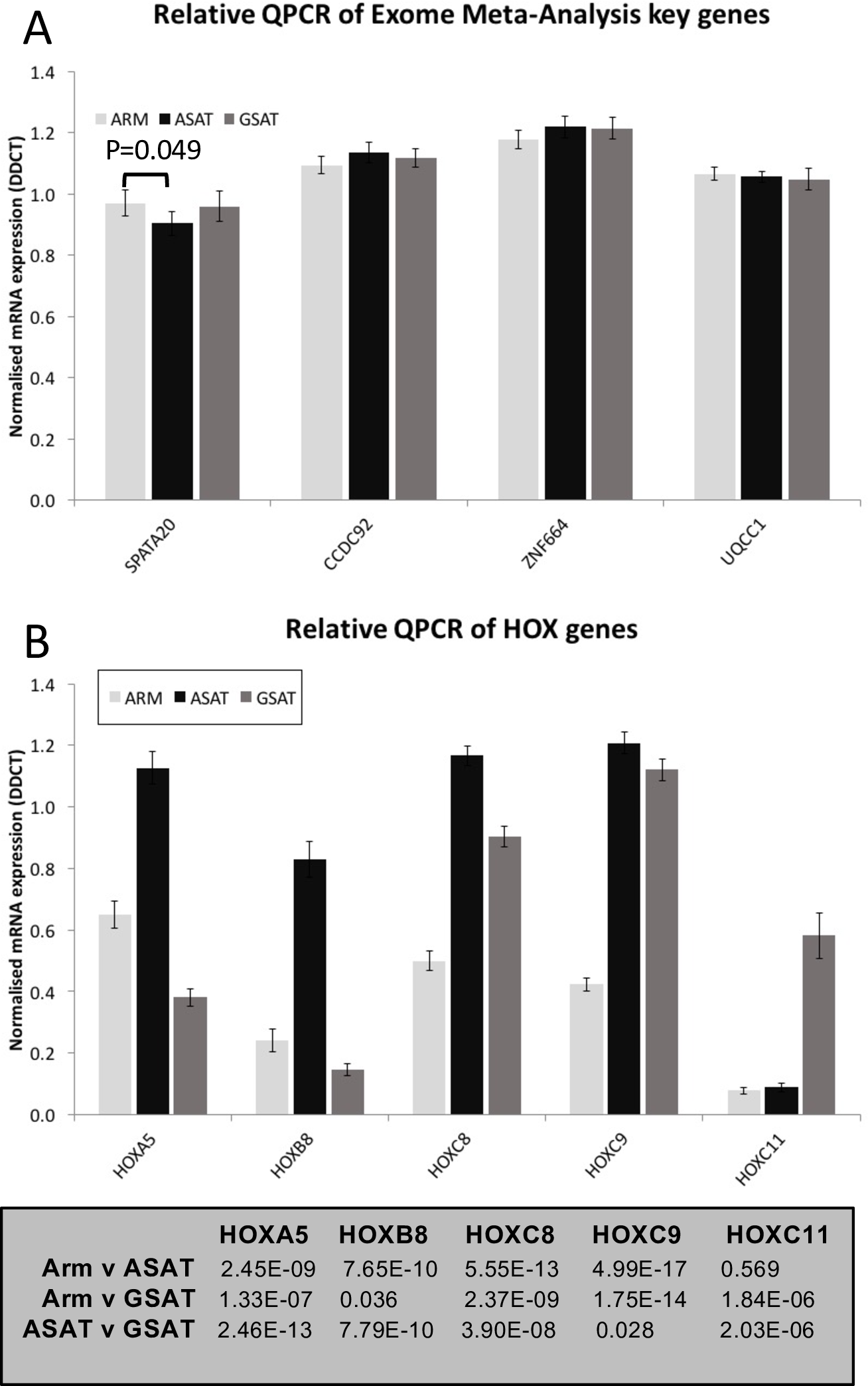

Supplement: S2 Fig — mRNA expression of the candidate genes A: CCDC92, ZNF664, UQCC1 and SPATA20 and a selection of developmental HOX genes B: HOXA5, HOXB8, HOXC8, HOXC9 and HOXC11 were determined by real-time qPCR. Data are shown as the mean ±SEM DDCt values (normalized to PPIA, PGK1 and PSMB6; n = 22). A univariate general linear model was used to test for statistical significance between depots. P-values for the HOX genes in B are presented in the shaded box. (TIF) [file pone.0217644.s002.tif]

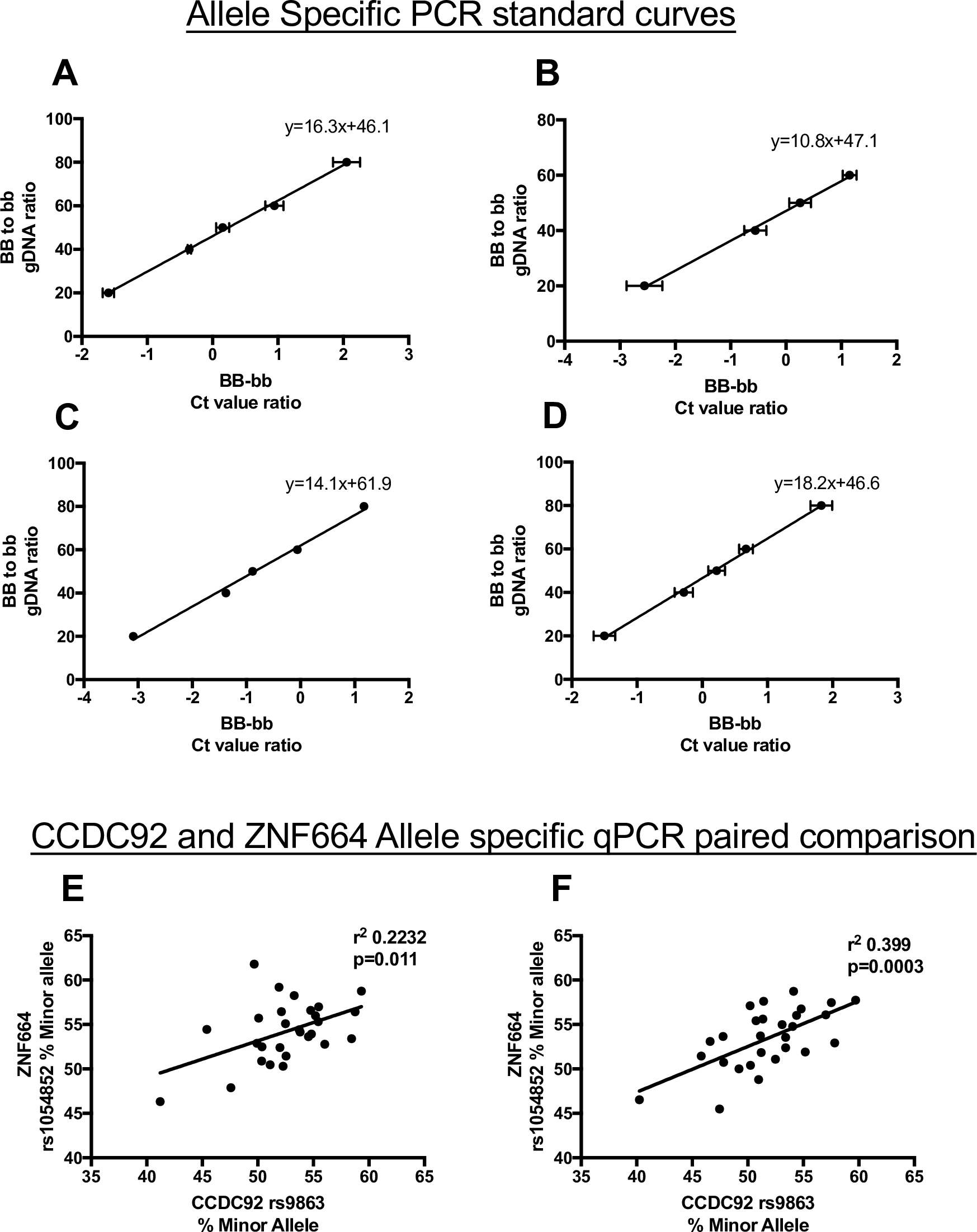

Supplement: S3 Fig — The standard curve and regression statistic used to calculate the percentage minor allele expression with allele-specific qPCR is shown above for CCDC92 (A), ZNF664 (B), SPATA20 (C) and UQCC1 (D). To quantify any allelic expression imbalance for the four genes a standard curve was generated from genomic DNA for individuals homozygous for the Major allele (BB) and Minor allele (bb). Genomic DNAs are diluted to 1.5ng/μl then BB and bb homozygotes were combined to ratios 80:20, 60:40, 50:50,40:60,80:20 to generate a standard curve. Following qPCR analysis using dual labelled TaqMan Genotyping assays the ratio of the B to b allele Ct values are calculated (Ct B minus Ct b) then plotted against the percentage of the minor allele in the dilution series. The linear regression statistic from this (A, B, C and D above) is then used to calculate the percentage minor allele expression of our unknown individuals. For CCDC92 (A), ZNF664 (B) and UQCC1 (D) three different pairs of homozygote individuals were used to generate each standard curve and a Mean ± SEM plotted for each dilution (A, B and D). For SPATA20 only one genomic DNA homozygote minor allele individual was available so an error bar cannot be displayed. As discussed in the main text there was an observed co-regulatory pattern of expression between CCDC92 and ZNF664 across different cDNA panels. To assess any correlation between these two genes within the samples, the allele-specific qPCR paired data points were plotted and regression statistic calculated (Graphs E and F). For both abdominal subcutaneous adipose tissue (ASAT) (E) and gluteal subcutaneous adipose tissue (GSAT) (F) there was a significant correlation, further supporting the co-regulatory pattern of expression. (TIF) [file pone.0217644.s003.tif]
