# Supplementary material for: Regional fat depot masses are influenced by protein-coding gene variants
Source: PLoS One. 2019 May 30;14(5):e0217644. doi: 10.1371/journal.pone.0217644 (PMC6542527; doi:10.1371/journal.pone.0217644)
Supplement: S1 Table — (DOCX) [file pone.0217644.s004.docx]

S1 Table. Population Cohort Descriptives

| **Study** | **Traits** | **Women** | | | **Men** | | |
| --- | --- | --- | --- | --- | --- | --- | --- |
|  |  | mean | SD | N | mean | SD | N |
| **Fenland-ExomeChip** | arm fat (g) | 2899 | 991 | 621 | 2372 | 673 | 524 |
|  | leg fat (g) | 10176 | 3634 | 621 | 7458 | 2154 | 524 |
|  | android fat (g) | 2179 | 1258 | 621 | 2582 | 1150 | 524 |
|  | gynoid fat (g) | 5000 | 1751 | 621 | 3731 | 1164 | 524 |
|  | visceral fat (g) | 629 | 578 | 600 | 1382 | 836 | 524 |
|  | subcutaneous fat (g) | 1550 | 788 | 621 | 1200 | 487 | 524 |
|  | total body fat % | 37 | 7 | 621 | 29 | 6 | 524 |
|  | age | 48 | 7 | 621 | 48 | 7 | 524 |
| **Fenland-CoreExome** | arm fat (g) | 2913 | 897 | 573 | 2379 | 719 | 462 |
|  | leg fat (g) | 9965 | 3082 | 573 | 7285 | 2220 | 462 |
|  | android fat (g) | 2134 | 1185 | 573 | 2490 | 1125 | 462 |
|  | gynoid fat (g) | 4881 | 1506 | 573 | 3634 | 1197 | 462 |
|  | visceral fat (g) | 599 | 562 | 551 | 1320 | 800 | 461 |
|  | subcutaneous fat (g) | 1535 | 760 | 573 | 1170 | 546 | 462 |
|  | total body fat % | 37 | 7 | 573 | 29 | 6 | 462 |
|  | age | 51 | 7 | 573 | 52 | 7 | 462 |
| **Fenland-Axiom** | arm fat (g) | 2773 | 1127 | 3912 | 2249 | 883 | 3531 |
|  | leg fat (g) | 9743 | 4165 | 3912 | 7066 | 2787 | 3531 |
|  | android fat (g) | 2042 | 1263 | 3912 | 2431 | 1286 | 3531 |
|  | gynoid fat (g) | 4765 | 1975 | 3912 | 3539 | 1440 | 3531 |
|  | visceral fat (g) | 577 | 548 | 3739 | 1299 | 867 | 3526 |
|  | subcutaneous fat (g) | 1465 | 815 | 3912 | 1132 | 606 | 3531 |
|  | total body fat % | 35 | 12 | 3912 | 27 | 10 | 3531 |
|  | age | 49 | 7 | 3912 | 49 | 7 | 3531 |
| **EPIC-Norfolk** | arm fat (g) | 3137 | 995 | 1706 | 2436 | 692 | 1395 |
|  | leg fat (g) | 9354 | 2954 | 1706 | 6513 | 1847 | 1395 |
|  | android fat (g) | 2284 | 1081 | 1706 | 2646 | 1072 | 1395 |
|  | gynoid fat (g) | 4585 | 1429 | 1706 | 3443 | 1073 | 1395 |
|  | visceral fat (g) | 886 | 602 | 1702 | 1771 | 899 | 1395 |
|  | subcutaneous fat (g) | 1396 | 613 | 1705 | 876 | 408 | 1394 |
|  | total body fat % | 39 | 6 | 1706 | 30 | 6 | 1395 |
|  | age | 54 | 7 | 1706 | 54 | 7 | 1395 |
| **Oxford Biobank Exome Chip** | arm fat (g) | 2806 | 1101 | 1848 | 2289 | 890 | 1433 |
|  | leg fat (g) | 9144 | 3315 | 1848 | 6317 | 2270 | 1433 |
|  | android fat (g) | 1935 | 1215 | 1848 | 2283 | 1197 | 1433 |
|  | gynoid fat (g) | 4603 | 1584 | 1848 | 3480 | 1242 | 1433 |
|  | visceral fat (g) | 456 | 480 | 1840 | 1157 | 799 | 1428 |
|  | subcutaneous fat (g) | 1463 | 779 | 1840 | 1109 | 523 | 1428 |
|  | total body fat % | 35 | 7 | 1848 | 27 | 7 | 1433 |
|  | age | 41 | 6 | 1848 | 42 | 6 | 1433 |
| **Oxford Biobank Axiom** | arm fat (g) | 2756 | 1041 | 777 | 2390 | 815 | 548 |
|  | leg fat (g) | 9076 | 3178 | 777 | 6524 | 2391 | 548 |
|  | android fat (g) | 1790 | 1173 | 777 | 2158 | 1148 | 548 |
|  | gynoid fat (g) | 4465 | 1559 | 777 | 3420 | 1252 | 548 |
|  | visceral fat (g) | 390 | 440 | 774 | 1039 | 757 | 548 |
|  | subcutaneous fat (g) | 1383 | 769 | 774 | 1119 | 551 | 548 |
|  | total body fat % | 35 | 8 | 777 | 27 | 7 | 548 |
|  | age | 41 | 6 | 777 | 41 | 6 | 548 |
