# Supplementary material for: Regional fat depot masses are influenced by protein-coding gene variants
Source: PLoS One. 2019 May 30;14(5):e0217644. doi: 10.1371/journal.pone.0217644 (PMC6542527; doi:10.1371/journal.pone.0217644)
Supplement: S2 Table — Detailed data on the three exome-wide significant loci described. DXA parameters are included for all measures and meta-analysis statistics for the additive model. DXA measures are arm fatmass (Arm), Total android fat mass (Android), Subcutaneous android fat mass (Subcut), Visceral android fat mass (Visceral), Gluteal fat mass (Gluteal) and Leg fat mass (Leg). Effect size data for suggestive exome-wide significance (p< = 10−6) is shown in bold. Exome-wide significant data (p<2E-7) are in bold and underlined. a The impact of missense variants were assessed using the PREDICTsnp online consensus tool[13] (https://loschmidt.chemi.muni.cz/predictsnp1/). b Approximate fat mass (grams) changes per allele is shown where test reaches suggestive significance and were calculated as marginal means after adjusting for age, PCs1-4 and %fatmass as covariates in a general linear model, implemented in SPSS v24 (DOCX) [file pone.0217644.s005.docx]

S2 Table. Exome-wide significant loci

| Chr:Position (GRCH37) | rsID | Gene | Effect | Transcript | PREDICTsnp ^a^ | DXA Measure | Gender | N | Pooled  EAF | Effect Size | Effect Size SE | P | Approximate change  per allele Raw Data^b^ | Approximate change  per allele Log Data^b^ |
| --- | --- | --- | --- | --- | --- | --- | --- | --- | --- | --- | --- | --- | --- | --- |
| 12:124427306 | rs11057401 | CCDC92 | S70C | NM_025140 | Deleterious | Arm | All | 17184 | 0.321 | -0.021 | 0.012 | NS |  |  |
|  |  |  |  |  |  | Arm | Men | 7831 | 0.323 | -0.014 | 0.017 | NS |  |  |
|  |  |  |  |  |  | Arm | Women | 9353 | 0.319 | -0.025 | 0.016 | NS |  |  |
|  |  |  |  |  |  |  |  |  |  |  |  |  |  |  |
|  |  |  |  |  |  | **ASAT** | **All** | **17184** | **0.321** | **-0.065** | **0.012** | **3.5E-08** | **-22g (-2.2%)** | **-30g (-3.4%)** |
|  |  |  |  |  |  | **ASAT** | **Men** | **7831** | **0.323** | **-0.079** | **0.017** | **5.5E-06** | **-23g (-2%)** | **-26g (-2.7%)** |
|  |  |  |  |  |  | ASAT | Women | 9353 | 0.319 | -0.055 | 0.016 | 5.7E-04 |  |  |
|  |  |  |  |  |  |  |  |  |  |  |  |  |  |  |
|  |  |  |  |  |  | Subcut | All | 17154 | 0.321 | -0.014 | 0.012 | NS |  |  |
|  |  |  |  |  |  | Subcut | Men | 7820 | 0.323 | 0.015 | 0.017 | NS |  |  |
|  |  |  |  |  |  | Subcut | Women | 9334 | 0.319 | -0.037 | 0.016 | 2.2E-02 |  |  |
|  |  |  |  |  |  |  |  |  |  |  |  |  |  |  |
|  |  |  |  |  |  | **Visceral** | **All** | **16967** | **0.321** | **-0.063** | **0.012** | **1.3E-07** | **-24g (-6.4%)** | **-20g (-9.9%)** |
|  |  |  |  |  |  | **Visceral** | **Men** | **7820** | **0.323** | **-0.088** | **0.017** | **3.7E-07** | **-37g (-6.4%)** | **-61g (-13.8%)** |
|  |  |  |  |  |  | Visceral | Women | 9147 | 0.319 | -0.042 | 0.016 | 9.4E-03 |  |  |
|  |  |  |  |  |  |  |  |  |  |  |  |  |  |  |
|  |  |  |  |  |  | GSAT | All | 17184 | 0.321 | 0.05 | 0.012 | 2.7E-05 |  |  |
|  |  |  |  |  |  | GSAT | Men | 7831 | 0.323 | 0.056 | 0.017 | 1.2E-03 |  |  |
|  |  |  |  |  |  | GSAT | Women | 9353 | 0.319 | 0.047 | 0.016 | 3.3E-03 |  |  |
|  |  |  |  |  |  |  |  |  |  |  |  |  |  |  |
|  |  |  |  |  |  | **Leg** | **All** | **17184** | **0.321** | **0.075** | **0.012** | **4.9E-10** | **95g (2.4%)** | **92g (2.5%)** |
|  |  |  |  |  |  | Leg | Men | 7831 | 0.323 | 0.076 | 0.017 | 1.2E-05 |  |  |
|  |  |  |  |  |  | **Leg** | **Women** | **9353** | **0.319** | **0.075** | **0.016** | **2.8E-06** | **120g (2.7%)** | **108g (2.5%)** |

| Chr:Position (GRCH37) | rsID | Gene | Effect | Transcript | PREDICTsnp ^a^ | DXA Measure | Gender | N | Pooled  EAF | Effect Size | Effect Size SE | P | Approximate change  per allele Raw Data^b^ | Approximate change  per allele Log Data^b^ |
| --- | --- | --- | --- | --- | --- | --- | --- | --- | --- | --- | --- | --- | --- | --- |
| 17:48628160 | rs62621401 | SPATA20 | K422R | NM_022827 | Neutral | **Arm** | **All** | **17204** | **0.016** | **-0.293** | **0.043** | **1.4E-11** | **-138g (-5.4%)** | **-87g (-3.6%)** |
|  |  |  | K406R | NM_001258372 |  | Arm | Men | 7844 | 0.015 | -0.291 | 0.066 | 1.0E-05 |  |  |
|  |  |  | K362R | NM_001258373 |  | **Arm** | **Women** | **9360** | **0.016** | **-0.296** | **0.057** | **2.5E-07** | **-105g (-3.8%)** | **-67g (-2.6%)** |
|  |  |  |  |  |  |  |  |  |  |  |  |  |  |  |
|  |  |  |  |  |  | ASAT | All | 17204 | 0.016 | 0.093 | 0.043 | 3.2E-02 |  |  |
|  |  |  |  |  |  | ASAT | Men | 7844 | 0.015 | 0.082 | 0.066 | NS |  |  |
|  |  |  |  |  |  | ASAT | Women | 9360 | 0.016 | 0.104 | 0.057 | NS |  |  |
|  |  |  |  |  |  |  |  |  |  |  |  |  |  |  |
|  |  |  |  |  |  | Subcut | All | 17174 | 0.016 | 0.081 | 0.043 | NS |  |  |
|  |  |  |  |  |  | Subcut | Men | 7833 | 0.015 | 0.039 | 0.066 | NS |  |  |
|  |  |  |  |  |  | Subcut | Women | 9341 | 0.016 | 0.106 | 0.057 | NS |  |  |
|  |  |  |  |  |  |  |  |  |  |  |  |  |  |  |
|  |  |  |  |  |  | Visceral | All | 16987 | 0.016 | 0.051 | 0.044 | NS |  |  |
|  |  |  |  |  |  | Visceral | Men | 7833 | 0.015 | 0.069 | 0.066 | NS |  |  |
|  |  |  |  |  |  | Visceral | Women | 9154 | 0.016 | 0.042 | 0.058 | NS |  |  |
|  |  |  |  |  |  |  |  |  |  |  |  |  |  |  |
|  |  |  |  |  |  | GSAT | All | 17204 | 0.016 | 0.06 | 0.043 | NS |  |  |
|  |  |  |  |  |  | GSAT | Men | 7844 | 0.015 | 0.036 | 0.066 | NS |  |  |
|  |  |  |  |  |  | GSAT | Women | 9360 | 0.016 | 0.076 | 0.057 | NS |  |  |
|  |  |  |  |  |  |  |  |  |  |  |  |  |  |  |
|  |  |  |  |  |  | Leg | All | 17204 | 0.016 | -0.008 | 0.043 | NS |  |  |
|  |  |  |  |  |  | Leg | Men | 7844 | 0.015 | -0.04 | 0.066 | NS |  |  |
|  |  |  |  |  |  | Leg | Women | 9360 | 0.016 | 0.021 | 0.057 | NS |  |  |

| Chr:Position (GRCH37) | rsID | Gene | Effect | Transcript | PREDICTsnp ^a^ | DXA Measure | Gender | N | Pooled  EAF | Effect Size | Effect Size SE | P | Approximate change  per allele Raw Data^b^ | Approximate change  per allele Log Data^b^ |
| --- | --- | --- | --- | --- | --- | --- | --- | --- | --- | --- | --- | --- | --- | --- |
| 20:33971914 | rs4911494 | UQCC1 | R51Q | NM_018244 | Neutral | **Arm** | **All** | **17197** | **0.616** | **-0.063** | **0.012** | **1.3E-07** | **-29g (-2.2%)** | **-35g (-2.9%)** |
|  |  |  | R51Q | NM_199487 |  | Arm | Men | 7839 | 0.616 | -0.071 | 0.017 | 2.7E-05 |  |  |
|  |  |  |  |  |  | Arm | Women | 9358 | 0.616 | -0.057 | 0.016 | 2.8E-04 |  |  |
|  |  |  |  |  |  |  |  |  |  |  |  |  |  |  |
|  |  |  |  |  |  | ASAT | All | 17197 | 0.616 | -0.03 | 0.012 | 1.3E-02 |  |  |
|  |  |  |  |  |  | ASAT | Men | 7839 | 0.616 | -0.036 | 0.017 | 3.5E-02 |  |  |
|  |  |  |  |  |  | ASAT | Women | 9358 | 0.616 | -0.028 | 0.016 | NS |  |  |
|  |  |  |  |  |  |  |  |  |  |  |  |  |  |  |
|  |  |  |  |  |  | Subcut | All | 17167 | 0.616 | -0.031 | 0.012 | 8.8E-03 |  |  |
|  |  |  |  |  |  | Subcut | Men | 7828 | 0.616 | -0.029 | 0.017 | NS |  |  |
|  |  |  |  |  |  | Subcut | Women | 9339 | 0.616 | -0.034 | 0.016 | 3.4E-02 |  |  |
|  |  |  |  |  |  |  |  |  |  |  |  |  |  |  |
|  |  |  |  |  |  | Visceral | All | 16980 | 0.616 | -0.007 | 0.012 | NS |  |  |
|  |  |  |  |  |  | Visceral | Men | 7828 | 0.616 | -0.021 | 0.017 | NS |  |  |
|  |  |  |  |  |  | Visceral | Women | 9152 | 0.615 | 0.002 | 0.016 | NS |  |  |
|  |  |  |  |  |  |  |  |  |  |  |  |  |  |  |
|  |  |  |  |  |  | GSAT | All | 17197 | 0.616 | -0.053 | 0.013 | 2.3E-05 |  |  |
|  |  |  |  |  |  | GSAT | Men | 7839 | 0.616 | -0.055 | 0.017 | 1.4E-03 |  |  |
|  |  |  |  |  |  | GSAT | Women | 9358 | 0.616 | -0.055 | 0.016 | 7.2E-04 |  |  |
|  |  |  |  |  |  |  |  |  |  |  |  |  |  |  |
|  |  |  |  |  |  | Leg | All | 17197 | 0.616 | -0.047 | 0.013 | 2.0E-04 |  |  |
|  |  |  |  |  |  | Leg | Men | 7839 | 0.616 | -0.057 | 0.017 | 9.7E-04 |  |  |
|  |  |  |  |  |  | Leg | Women | 9358 | 0.616 | -0.039 | 0.016 | 1.4E-02 |  |  |
