# Supplementary material for: Regional fat depot masses are influenced by protein-coding gene variants
Source: PLoS One. 2019 May 30;14(5):e0217644. doi: 10.1371/journal.pone.0217644 (PMC6542527; doi:10.1371/journal.pone.0217644)
Supplement: S3 Table — Additional non-synonymous loci where statistical tests did not reach exome-wide significance but did reach a suggestive significance cut off of p< = 10–6 are included above. a Where it reaches suggestive significance the model is shown as Additive (add), Recessive(rec) or Dominant (Dom). b The impact of missense variants were assessed using the predictSNP online consensus tool[13] (https://loschmidt.chemi.muni.cz/predictsnp1/). c The cluster of 8 Missense SNPs found at the SPPL2C-MAPT-KANSL1 locus on chromosome 17 are part of a single haplotype that extends across ~400kb in this region containing >2300 SNPs (r2>0.9), rather than independent signals. (DOCX) [file pone.0217644.s006.docx]

S3 Table. Exome-wide loci showing suggestive level of statistical significance

| **Chr:Position (GRCH37)** | **rsID** | **Gene** | **Amino Acid  change** | **Predicted  impact^b^** | **DXA Measure** | **Gender** | **Test^a^** | **Ref Allele** | **Alt Allele** | **N** | **Alt AF** | **Effect Size** | **Effect Size SE** | **P** |
| --- | --- | --- | --- | --- | --- | --- | --- | --- | --- | --- | --- | --- | --- | --- |
| 1:70625071 | rs145682711 | LRRC40 | P388S | Deleterious | subcut | all | add | G | A | 17163 | 0.003 | -0.479 | 0.103 | 3.2E-06 |
| 1:201016296 | rs3850625 | CACNA1S | R1539C | Deleterious | android | all | dom | G | A | 17211 | 0.119 | -0.084 | 0.019 | 5.0E-06 |
| 2:27730940 | rs1260326 | GCKR | L446P | Neutral | arm | all | add | T | C | 17153 | 0.593 | 0.057 | 0.011 | 5.2E-07 |
| 2:27730940 | rs1260326 | GCKR | L446P | Neutral | arm | women | add | T | C | 9339 | 0.592 | 0.073 | 0.015 | 2.1E-06 |
| 2:27730940 | rs1260326 | GCKR | L446P | Neutral | subcut | all | add | T | C | 17123 | 0.593 | 0.053 | 0.012 | 4.8E-06 |
| 2:61575149 | rs151293144 | USP34 | I714T | Neutral | gynoid | women | add | A | G | 9362 | 0.003 | -0.582 | 0.129 | 6.4E-06 |
| 3:44943389 | rs1995641 | TGM4 | E313K | Neutral | visceral | all | dom | G | A | 16966 | 0.411 | -0.079 | 0.016 | 1.7E-06 |
| 3:150391810 | rs73003074 | FAM194A | L426V | Neutral | leg | all | rec | A | C | 17074 | 0.137 | 0.258 | 0.058 | 8.0E-06 |
| 5:10239261 | rs2438652 | FAM173B | T75M | Neutral | android | women | add | G | A | 9361 | 0.133 | -0.103 | 0.022 | 2.1E-06 |
| 6:34214322 | rs1150781 | C6orf1 | G150A,G130A | Neutral | visceral | men | add | C | G | 7813 | 0.909 | -0.132 | 0.029 | 3.5E-06 |
| 6:130374102 | rs9388768 | L3MBTL3 | T158N,T183N | Neutral | gynoid | all | add | C | A | 17183 | 0.674 | -0.053 | 0.012 | 6.5E-06 |
| 6:130374102 | rs9388768 | L3MBTL3 | T158N,T183N | Neutral | leg | all | add | C | A | 17183 | 0.674 | -0.052 | 0.012 | 8.9E-06 |
| 8:11704617 | rs114308907 | CTSB | N246T | Deleterious | gynoid | men | add | T | G | 7835 | 0.001 | 1.024 | 0.23 | 8.2E-06 |
| 9:139405111 | rs201620358 | NOTCH1 | R912W | Deleterious | android | men | add | G | A | 7841 | 0.004 | 0.58 | 0.129 | 6.4E-06 |
| 10:27317840 | rs10829163 | ANKRD26 | V1305I | Neutral | android | all | dom | C | T | 17105 | 0.135 | 0.084 | 0.018 | 2.6E-06 |
| 10:69644820 | rs182199697 | SIRT1 | P114Q | Neutral | leg | all | add | C | A | 17196 | 0.007 | 0.283 | 0.064 | 8.8E-06 |
| 11:36615697 | rs150762709 | RAG2 | V8I | Neutral | subcut | women | rec | C | T | 9338 | 0.005 | 3.211 | 0.703 | 5.0E-06 |
| 11:68201295 | rs3736228 | LRP5 | A1330V | Neutral | leg | all | add | C | T | 17199 | 0.151 | -0.071 | 0.015 | 4.5E-06 |
| 11:68201295 | rs3736228 | LRP5 | A1330V | Neutral | leg | all | dom | C | T | 17199 | 0.151 | -0.079 | 0.017 | 5.1E-06 |
| 12:49491793 | rs141634230 | LMBR1L | L446F | Deleterious | subcut | men | add | G | A | 7828 | 0.001 | -1.223 | 0.249 | 9.2E-07 |
| 16:4445327 | rs3747579 | CORO7 | R175Q,R108Q,R193Q | Neutral | gynoid | all | add | C | T | 17177 | 0.714 | 0.056 | 0.012 | 6.7E-06 |
| 16:4445327 | rs3747579 | CORO7 | R175Q,R108Q,R193Q | Neutral | leg | women | add | C | T | 9345 | 0.715 | 0.073 | 0.016 | 9.1E-06 |
| 17:16842991 | rs34562254 | TNFRSF13B | P251L | Neutral | android | all | rec | G | A | 17186 | 0.101 | 0.338 | 0.074 | 4.7E-06 |
| 17:16842991 | rs34562254 | TNFRSF13B | P251L | Neutral | android | men | rec | G | A | 7829 | 0.1 | 0.517 | 0.113 | 4.3E-06 |
| 17:43922942 ^c^ | rs62621252 | SPPL2C | S224P | Neutral | visceral | women | add | T | C | 9086 | 0.24 | 0.09 | 0.019 | 1.5E-06 |
| 17:43923266 ^c^ | rs62054815 | SPPL2C | A332T | Neutral | visceral | women | add | G | A | 9153 | 0.237 | 0.093 | 0.019 | 6.8E-07 |
| 17:43923683 ^c^ | rs12185268 | SPPL2C | I471V | Neutral | visceral | women | add | A | G | 9136 | 0.238 | 0.093 | 0.019 | 7.3E-07 |
| 17:43924073 ^c^ | rs12373123 | SPPL2C | S601P | Neutral | visceral | women | add | T | C | 9153 | 0.237 | 0.093 | 0.019 | 7.0E-07 |
| 17:44061278 ^c^ | rs17651549 | MAPT | R370W | Deleterious | visceral | women | add | C | T | 9142 | 0.237 | 0.091 | 0.019 | 1.3E-06 |
| 17:44067400 ^c^ | rs10445337 | MAPT | S447P | Neutral | visceral | women | add | T | C | 9053 | 0.239 | 0.087 | 0.019 | 3.8E-06 |
| 17:44076665 ^c^ | rs62063857 | MAPT | Q7R | Neutral | visceral | women | add | A | G | 9151 | 0.237 | 0.091 | 0.019 | 1.2E-06 |
| 17:44117119 ^c^ | rs34043286 | KANSL1 | S718P | Neutral | visceral | women | add | A | G | 9142 | 0.237 | 0.09 | 0.019 | 1.6E-06 |
| 17:51900729 | rs3803824 | KIF2B | A112V | Neutral | leg | women | rec | C | T | 9348 | 0.415 | 0.126 | 0.028 | 5.8E-06 |
| 17:78272294 | rs72849841 | RNF213 | P729L | Neutral | leg | men | add | C | T | 7836 | 0.115 | -0.124 | 0.025 | 9.5E-07 |
| 18:43796123 | rs201155841 | C18orf25 | C93R | Deleterious | gynoid | women | add | T | C | 9363 | 0.003 | -0.588 | 0.128 | 4.2E-06 |
| 18:43796123 | rs201155841 | C18orf25 | C93R | Deleterious | leg | women | add | T | C | 9363 | 0.003 | -0.591 | 0.128 | 4.1E-06 |
| 20:34022387 | rs224331 | GDF5 | S276A | Neutral | arm | all | add | A | C | 17145 | 0.117 | 0.098 | 0.02 | 1.6E-06 |
| 20:34022387 | rs224331 | GDF5 | S276A | Neutral | arm | women | add | A | C | 9326 | 0.119 | 0.132 | 0.027 | 9.0E-07 |
